# Pilot-scale bioethanol production from the starch of avocado seeds using a combination of dilute acid-based hydrolysis and alcoholic fermentation by Saccharomyces cerevisiae

**DOI:** 10.1186/s12934-023-02110-5

**Published:** 2023-06-29

**Authors:** Luis Caballero-Sanchez, Pedro E. Lázaro-Mixteco, Alejandra Vargas-Tah, Agustín J. Castro-Montoya

**Affiliations:** 1 Posgrado de Ingeniería Química, Universidad Michoacana de San Nicolás de Hidalgo, Francisco J. Múgica S/N, Ciudad Universitaria, 58030 Morelia, Mich México; 2 Facultad de Ingeniería Química, Universidad Michoacana de San Nicolás de Hidalgo, Francisco J. Múgica S/N, Ciudad Universitaria, 58030 Morelia, Mich México

**Keywords:** Bioethanol production, Avocado seeds, Starch, Hydrolysis, Fermentation

## Abstract

**Background:**

A processing methodology of raw starch extraction from avocado seeds (ASs) and a sequential hydrolysis and fermentation bioprocess in just a few steps was successfully obtained for the bioethanol production by a single yeast *Saccharomyces cerevisiae* strain and this research was also to investigate the optimum conditions for the pretreatment of biomass and technical procedures for the production of bioethanol. It successfully resulted in high yields and productivity of all the experiments from the laboratory scale and the pilot plant. Ethanol yields from pretreated starch are comparable with those in commercial industries that use molasses and hydrolyzed starch as raw materials.

**Results:**

Before the **pilot-scale bioethanol** production, studies of starch extraction and dilute sulfuric acid-based pretreatment was carefully conducted. The amount of starch extracted from dry and fresh avocado seed was 16.85 g ± 0.34 g and 29.79 ± 3.18 g of dry starch, representing a yield of ∼17% and 30%, respectively. After a dilute sulfuric acid pretreatment of starch, the released reducing sugars (RRS) were obtained and the hydrolysate slurries containing glucose (109.79 ± 1.14 g/L), xylose (0.99 ± 0.06 g/L), and arabinose (0.38 ± 0.01 g/L). The efficiency of total sugar conversion was 73.40%, with a productivity of 9.26 g/L/h. The ethanol fermentation in a 125 mL flask fermenter showed that *Saccharomyces cerevisiae* (Fali, active dry yeast) produced the maximum ethanol concentration, *p*_max_ at 49.05 g/L (6.22% v/v) with a yield coefficient, *Y*_*p/s*_ of 0.44 g_Ethanol/_g_Glucose_, a productivity or production rate, *r*_*p*_ at 2.01 g/L/h and an efficiency, Ef of 85.37%. The pilot scale experiments of the ethanol fermentation using the 40-L fermenter were also successfully achieved with essentially good results. The values of *p*_max,_*Y*_*p/s*_, *r*_*p*_, and Ef of the 40-L scale were at 50.94 g/L (6.46% v/v), 0.45 g_Ethanol/_g_Glucose_, 2.11 g/L/h, and 88.74%, respectively. Because of using raw starch, major by-products, i.e., acetic acid in the two scales were very low, in ranges of 0.88–2.45 g/L, and lactic acid was not produced, which are less than those values in the industries.

**Conclusions:**

The sequential hydrolysis and fermentation process of two scales for ethanol production using the combination of hydrolysis by utilizing dilute sulfuric acid-based pretreatment and fermentation by a single yeast *Saccharomyces cerevisiae* strain is practicable and feasible for realistic and effective scale-up strategies of bioethanol production from the starch of avocado seeds.

## Introduction

Starch is a polymer of glucose and mainly consists of amylose and amylopectin. It is the second most important and abundant source of carbon and energy in a large variety of higher plants [1]. Starch is also a very important feedstock and it has a big demand in the industry to produce many valuable products, such as maltose, glucose, fructose, glucose-fructose syrups, organic acids, amino acids, etc. [2, 3]. Furthermore, starch is also an important feedstock in the fermentation industry, where is saccharified and fermented to produce ethanol, which can be employed for the production of biofuel, potable alcohols, e.g., beverages such as beer, whiskey, and other ethanol products [4]. The production of bioethanol from starch was first introduced at the beginning of the twentieth century as an alternative energy source to replace the utilization of conventional fuels [5, 6]. Bioethanol has a great advantage over conventional fuels: It has a higher octane rating and it is safer to use. It is also an eco-friendly renewable resource that contributes to the reduction of petroleum-based fuel emissions [6–9]. Nowadays, there are policies for blending 20–30% ethanol in gasoline in different countries by 2030. However, the availability of enough ethanol is still a challenge for this purpose, because agroindustrial residues, forest residues, etc. would also generate only a limited amount of bioethanol [10–12]. So, there is a need to explore the use of other wastes such as fruit wastes or vegetable wastes which are consumed at huge scales. In fact, every fruit generates 50% of its weight as waste after its consumption, which is a huge amount and is utilized to generate bioethanol [13].

Bioethanol is mainly obtained from corn starch and sugarcane [14]. However, can also be produced from various kinds of feedstock such as cassava, sugar beet, sweet potato, wheat, rice, and sorghum. All of these materials are made up of starch, which depending on the botanical source, contains certain amounts of amylose and amylopectin [4, 5, 14]. Interestingly, starch can also be obtained from unconventional sources such as avocado wastes [15]. The by-products from the avocado are mainly the peel (APs) and the seed (ASs), representing between 20 and 30 wt % of the fruit, which are often discarded or used as compost [16]. These two residues are rich in carbohydrates such as cellulose, hemicellulose, and starch and have a high potential for the production of value-added materials [17]. Specifically, the chemical composition of seed on Hass and Fuerte varieties is reported 2.4 and 2.5% protein, 3.5 and 2.2% sugar, 2.5 and 3.2% neutral lipids, 12 and 13% glycolipids, 7.4 and 10.9% phospholipids, 0.8 and 1.0% fat, respectively [18–20]. Starch represents nearly 60% of the seed (dry matter basis), resulting in large amounts of potentially fermentable sugars [21–23]. Consequently, avocado seeds stand out as promising feedstock for applications within industrial bioprocesses and the biorefinery concept for ethanol production and other bioproducts of commercial interest [22–25]. Importantly, in Mexico, Michoacan contributes on 75.2% (1,800,021 tons) to the national total production, followed by Jalisco with 10.4% (248,392 tons). These two adjoining states concentrate 85.6% (2,048,413 tons) of the country’s production [26], which would be a great opportunity to take the avocado by-products for its exploitation to generate bioethanol at a low cost.

A few studies have demonstrated that using avocado seed wastes can be saccharified and fermented with bacterial strains in a laboratory, pilot scale, and semi-industrial levels process to produce bioproducts with successful results [22, 25, 27–30]. In addition, for hydrolysis and fermentation of starch to bioethanol production, large companies have developed novel and efficient enzymes for the saccharification of starch [6]. However, the conventional enzymatic liquefaction and saccharification of starch have disadvantages in two main ways. First, they require enormous amounts of efficient water-based cooling systems to regulate the temperatures during fermentations, thus increasing the complexity, time, and production costs of starch-based ethanol [31]. Second, inhibitory effects in enzymatic activity may occur during the liquefaction or saccharification stages owing to high concentrations of starch or glucose present that act as a competitive inhibitor of the process [32–34].

Alternatively, the direct hydrolysis of raw starch to glucose with the dilute acid pretreatment (DAP) could significantly simplify processing and reduces the cost of producing starch-based products e.g., bioethanol and other bioproducts [3, 23]. This process could save on energy costs, as well as the total capital and operational costs [24, 35]. Through this process, the conversion of the complex carbohydrate content in starch into simple sugar forms is achievable through hydrolysis, by adding dilute acid (DA) in water molecules in the non-severe condition of temperature to separate the chain of starch [14, 36, 37]. In a series of bioethanol-production steps, the simple sugar units are highly required because the metabolism performed by microorganisms in the fermentation stage cannot be carried out with complex sugars [38]. So, the quality of the hydrolyzed starch is important to produce high ethanol concentrations. To hydrolysis these raw materials to sugars, diluting acid at high temperature is also one of the milder methods used to break down the complex carbohydrates [39], 90% yield of monosaccharides would be achieved [40]. It helps primarily in the partial solubilization of biomass after pretreatment increasing the digestibility by microbial metabolism [41]. It is very documented that acid hydrolysis is much faster and cheaper compared to the enzymatic method [40–42]. After pretreatment of raw starch, yeast strains such as *Saccharomyces cerevisiae*, *Scheffersomyces* (Pichia) *stipites*, *Kluyveromyces marxianus, Pachysolen tannophilus*, and *Candida shehatae* can assimilate and ferment sugars derived from hydrolysis of starch [43–45]. There are few reports of natural yeast strains that can yield an amylolytic enzyme and simultaneously produce efficiently ethanol from starch [46–48]. Therefore, researchers have improved the expression of amylolytic enzymes by using genetically engineered yeasts [49–54]. However, the use of such yeasts is associated with regulations, increasing the production costs, because a genetic-control project using a sterilization system and special laboratory enclosure is required to confine them, in order to limit the survival and their escaping and spread of the yeasts into the environment [55, 56]. For these microbial bioprocesses, the composition of the culture media is also an important factor, because it represents the nutrient source for growth and the production of metabolites of commercial interest [57]. So, the quality and cost of the culture media impact the global efficiency and economy of bioprocess, representing a bottleneck cost is the substrate [22].

Therefore, finding not only cheap and renewable feedstocks but also the high availability of this starchy material in Mexico, such as avocado residues, represents a field of opportunity in the industrial fermentation process of agroindustrial residues for microbial bioproducts. So, more research and development in pilot scale and semi-industrial levels for starch hydrolysis by diluted acid and subsequent sugar fermentation to bioethanol in just a few steps for bioprocessing of renewable lignocellulose biomass using natural yeast strains are required in order to render the process even more cost-effective. The objective of this research was to obtain a processing methodology for raw starch extraction from avocado seeds for sequential hydrolysis and ethanol fermentation. The second objective of this study was also to obtain a starch hydrolysate by a dilute acid-based pretreatment method for raw starch conversion to ethanol by fermentation using the yeast *Saccharomyces cerevisiae* without additional nutrient supply into hydrolysate, less time-consuming and lower operating cost at the laboratory scale and the 40-L pilot plant.

## Materials and methods

### Characterization of avocado seeds and starch extraction

The Hass-type avocado seed was provided by the SIMPLOT company (Morelia, Michoacan, Mexico). The chemical characterization of avocado seeds was performed according to the procedures described by the National Renewable Energy Laboratory (NREL). The analysis included the determination of moisture content [58], extractives [59], structural carbohydrates and lignin [60], and ash [61]. For starch isolation, the extraction was done in triplicate, using fresh and dried avocado seeds separately by adapting the methodology proposed by de Castro and coworkers [62], with modifications. The seeds were washed with tap water and finally with distilled water before cutting them into small pieces and dried in an oven at 80 °C for 24 h. Dried seeds were powdered in a grinder type 1RF3 054-4YC31 to reduce the particle size by passing it through a 40-micron sieve (425 μm) and then were retained in a 60-mesh sieve (250 μm). For the starch extraction process, a sample of 100 g of powdered seeds was immersed in 300 mL of distilled water, then ground in a food processor (Oster, 6805-RG0), and finally filtered through a cloth sieve (cotton fabric). The suspension obtained was left to stand for 24 h to complete the starch sedimentation. After the supernatant was discarded, the starchy pellets obtained were centrifuged at 4,000 rpm for 10 min and dried in an oven (NOVATECH, Model HS35-AID) for 24 h at 60 °C, ground, and stored at room temperature in a hermetically sealed container for further treatment. The yield of starch extraction process was calculated using Eq. (1):

$$Yield\left(\%\right)=\left(\frac{FMS}{MASs}\right)x100$$

where: FMS: Final mass starch (g); MASs: Mass of avocado seeds used in the extraction (g).

### Thermochemical hydrolysis of avocado starch in laboratory and pilot scales

In this study, a mixture of 100 g containing 15% w/w of starch powder of avocado seed and the balance with diluted acid 2% (w/w) H_2_SO_4_. The reaction of hydrolysis was carried out in an Erlenmeyer flask of 250 ml at 87 °C for 12 h at 100 rpm with a magnetic stirrer in a water bath using a cryothermostat (JULABO, model CORIO CP 200 F). The experiment was carried out in triplicate.

After the pretreatment procedure, starch hydrolysates were used as nutritious media to obtain bioethanol. Also, 1 mL aliquots of hydrolysate were centrifuged at 13,500 rpm (Fisher Scientific, accuSpin Micro 17) and the supernatants were analyzed for glucose, xylose, and arabinose content using high-performance liquid chromatography (HPLC). Released Reducing sugars (RRS) were characterized by the 3,5-dinitro salicylic acid method (DNS) before and after mild thermal-acid hydrolysis pretreatment.

### Fermentation of Starch hydrolysate of avocado seeds

For fermentation on the laboratory scale, the 125 mL shake-flask fermenters were utilized and 100 mL of the raw hydrolysate was used as a cultivation medium. 0.5 g of lyophilized yeast *Saccharomyces cerevisiae* (DistilaMax® DS strain) was hydrated in YPD medium at 35 °C for 30 min and directly inoculated into the starch hydrolysate with an initial inoculum concentration of 5 g/L. Fermentation conditions were: 30 °C, 50 rpm, and pH 5.0 adjusted with the addition of 14.6% NH_4_OH (v/v). All experiments were carried out in triplicate. The scale-up of dilute acid-based hydrolysis of starch from avocado seeds for ethanol fermentation was performed from the 125 mL shake-flask cultures until the pilot scale (PIGNAT, Model UPB/2000/S). The pilot plant contains a 40-L fermenter, which has a jacket that allows temperature control with steam, as well as a variable revolution impeller stirrer that can stir up to 125 rpm.

In this pretreatment scale, the fermenter contained 40 Kg of the fermentation mixture containing 15% w/w of starch powder of avocado seed and diluted acid 2% H_2_SO_4_(w/w). The mild pretreatment condition was: 87 °C and 30 rpm for 12 h. After hydrolysis, the pilot-scale ethanol fermentation was carefully conducted directly into the starch hydrolysate in the 40-L fermenter with an initial inoculum concentration of 5 g/L *Saccharomyces cerevisiae* strain. Fermentation conditions were: 30 °C, 30 rpm, and pH 5.0 adjusted with the addition of 14.6% NH_4_OH (v/v). The pilot plant experiments were carried out in duplicate.

### Fermentation in shake flask and pilot plant

Ethanol production kinetics were performed to determine the parameters of hydrolyzed slurries from the starch of avocado seeds were utilized as culture media. 1 mL aliquots were taken over the course of 24 h. For each sample, the supernatant was recovered and the concentrations of sugars, ethanol, and acetic acid were determined by HPLC. The experiments were carried out in triplicate substrate and product analysis.

## Results and discussion

### Characterization of avocado seeds

The Hass-type avocado seed provided by the SIMPLOT Company located in Morelia, Michoacan, Mexico, was characterized by its potential for use as feedstock for bioethanol production. The results of Hass seeds characterization on a dry basis (%w/w) presented in Table 1 show that the total carbohydrate in the seed is 58.51 g/100 g, which corresponds to cellulose 53.62 ± 1.72 and hemicellulose 4.89 ± 0.14. This value was slightly higher than 54.36 g/100 g compared to the published values previously reported [23, 63–65]. As carbohydrates are related to energy generation, this suggested that the biomass composition of avocado seeds dispose of enough carbohydrates to produce glucose as the carbon source for the growing microbial cells on consumption during the fermentation processes to produce value-added products such as bioethanol. The content of extractives in the seeds was 25.92 ± 1.31 and lignin 3.23 ± 0.63; these values are similar to those reported by other research groups (see Table 1).

**Table 1:** The characterization of avocado seed reported in this study and by different researchers

Composition	Proximate (%)	[63]	[64]	[65]	[23]
Moisture	11.69 ± 0.04	7.02 ± 0.18	13.17 ± 0.09	------	------
Extractives	25.92 ± 1.31	35.95 ± 1.95	26.45 ± 2.52	20.90	21.00–35.90
Cellulose	53.62 ± 1.72	6.48 ± 0.38	13.38 ± 0.45	37.00	6.50–40.90
Hemicellulose	4.89 ± 0.14	47.88 ± 2.14	9.30 ± 0.21	3.50	3.00–47.90
Lignin	3.23 ± 0.63	1.79 ± 0.04	7.78 ± 0.98	15.30	1.80–15.80
Ash	0.41 ± 0.02	0.87 ± 0.06	2.86 ± 0.10	2.76	0.90–2.90
Total	99.76	100	------	------	------

### Yield of starch extraction from avocado seeds and characterization of starch hydrolysate

As shown in Table 2, the amount of starch extracted from Hass avocado seed was 29.79 g of dry starch obtained from 100 g of sample, representing a yield of 29.79%. Silva et al. (2013) reported a starch yield of 11.36% with a difference of 18.43% from the present study [66]. Ginting et al. (2015) reported a starch yield of 24.20% with a difference of 5.59% from the present study [67]. Lubis et al. (2016) reported a starch yield of 16% (60 °C) which differs by 13.79% from the present study [68]. Kowalski et al. (2017) reported a starch yield of 20.10% with a difference of 9.69% from the resent study [69]. Correa et al. (2019) reported a starch yield of 6.85% which differs by 22. 94% with the presnt study [70]. Rosalia Jimenez et al. (2021)[15] reported a starch yield of 11.38% which differs by 18.4% from the present stud. Recently, Martins et al. (2022) reported a starch yield of 19.54, which differs by 7.25% from the present stud [20]. It is documented that factors that directly influence the yield of the starch extraction are extraction method, soil composition, and the avocado variety, which can directly affect the extraction results [20, 71]. So, the method used for the extraction of starch in the present work obtained a higher result and can be used for the next sequential steps of hydrolysis of the starch polymers to sugar units and fermentation for bioethanol production.

**Table 2:** The starch yield from avocado seed reported by different researchers

Entry	Reference	Year	Starch yield (%)	% of difference
1	This work	2023	29.79	-----
2	Martins et al. [20]	2022	19.54	7.25
3	Jimenez et al. [15]	2021	11.38	18.41
4	Correa et al. [70]	2019	6.88	22.91
5	Kowalski et al. [69]	2017	20.10	9.69
6	Lubis et al. [68]	2016	16.00	13.79
7	Ginting et al. [67]	2015	24.20	5.59
8	Silva et al. [66]	2013	11.36	18.43

### Hydrolysis of avocado seed starch

**Fig. 1:**
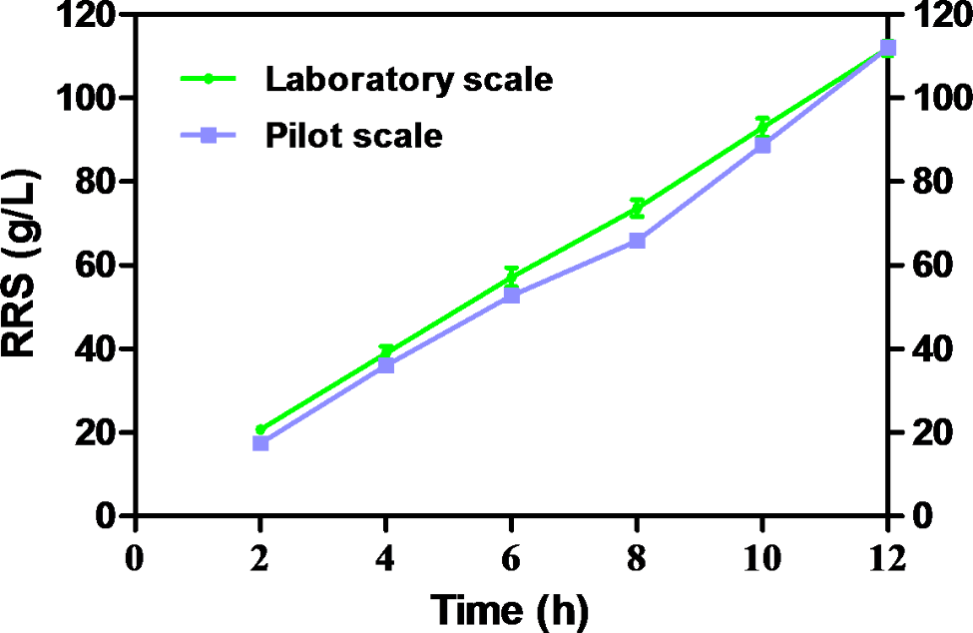
Effect of time on the release of reducing sugars from starch from avocado seed at laboratory and pilot scale

The pretreatment of raw starch was performed with diluted acid 2% (w/w) H_2_SO_4_ at 87 °C for 12 h. The analysis of the kinetics of glucose and RRS production was carried out for 12 h in the 250 mL flask and fermentation tank of the pilot plant (Fig. 1). The results show the average of the three replicates with the standard deviation. During this stage of hydrolysis, a significant increase in the RRS and glucose concentration is observed, until the end of the process. As for the concentrations of xylose and arabinose, their maximum values are observed at close to 12 h of processing. This value also corresponds to the maximum concentration of xylose reached during the entire hydrolysis process. The maximum concentration of RRS at laboratory and pilot scales were glucose (109.79 ± 1.14 g/L and 109.86 g/L, respectively), xylose (0.99 ± 0.06 g/L and 1.87 g/L, respectively), and arabinose (0.38 ± 0.01 g/L and 0.30 g/L, respectively). Therefore, this bioprocess can be technically quite easily feasible to scale up from 125 mL shake flasks to a 40-L pilot plant, reaching similar yields in both operational scales for fermentable sugars production.

### Ethanol fermentation by Saccharomyces cerevisiae at laboratory scale

**Fig. 2:**
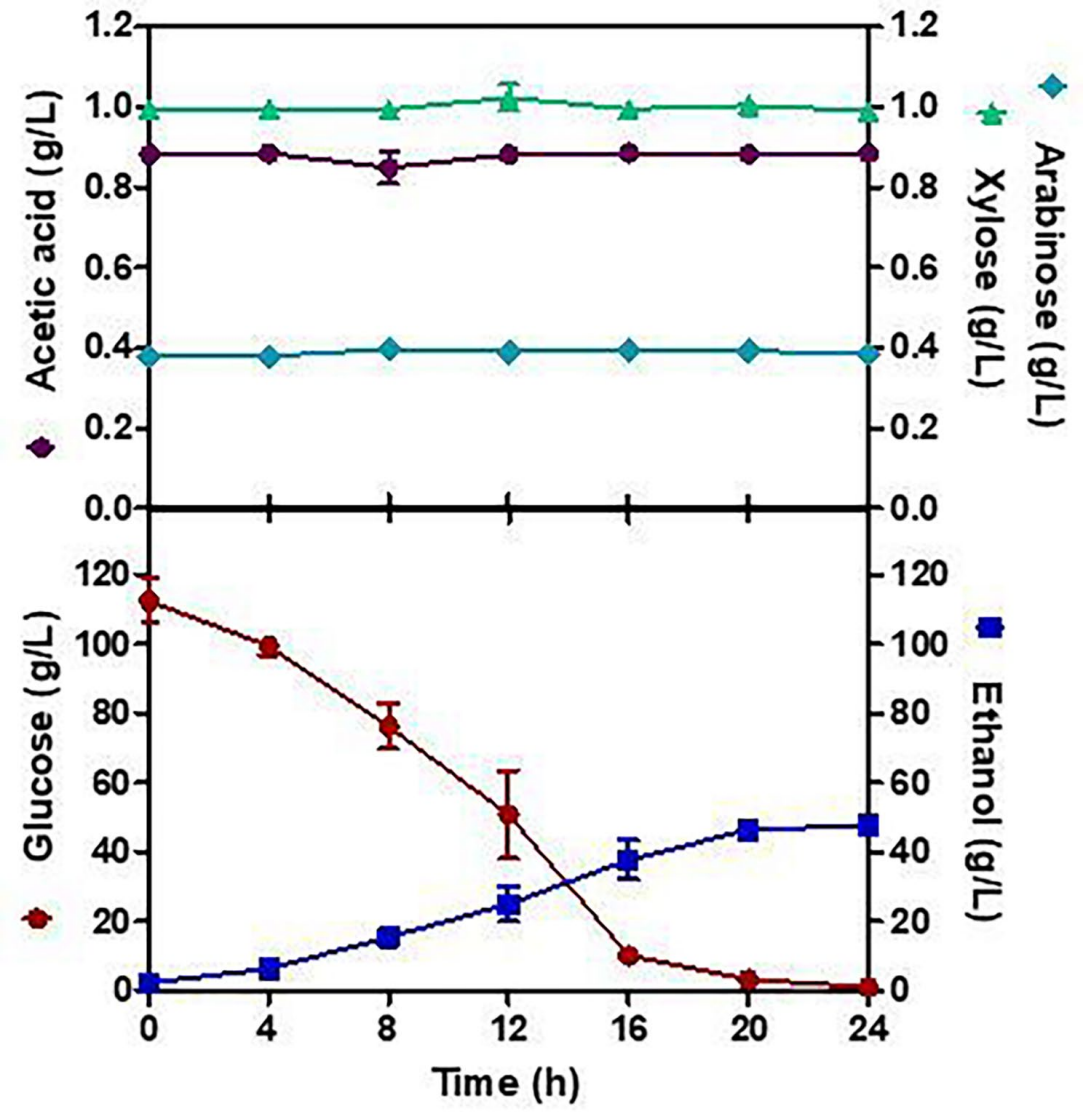
Fermentation kinetics of glucose obtained from the dilute acid pretreatment by *Saccharomyces cerevisiae* at laboratory scale

Studies on ethanol production at 30 ^o^C on a laboratory scale (125 mL Erlenmeyer flasks) using 100 mL of the starch hydrolysate without additional nutrient supply into hydrolysate were conducted under optimized fermentation conditions (The initial glucose concentration of 112.44 g/L, the initial inoculation size of 0.5% w/v, 30 ^o^C, and 50 rpm). Results presented in Fig. 2, show the average of the three replicates with the standard deviation, the fermentation development was also adjusted according to the desired condition at the pilot scale, a 24 h fermentation step. For that, hydrated yeast in the YPD medium had to be directly added at the beginning of fermentation, providing an initial concentration of 5 g cells/L on a dry basis. Figure 2 shows also that the ethanol concentration increases slightly over approximately the first 20 h of the fermentation process, reaching the maximum value of 49.05 g/L, and then levels off, with a yield coefficient, *Y*_*p/s*_ of 0.44 g_Ethanol/_g_Glucose_, a productivity or production rate, *r*_*p*_ at 2.01 g/L/h and an efficiency, Ef of 85.37%. The glucose degradation increased with fermentation time, and it was almost completely utilized after 20 h of fermentation. The exponential phase for sugar consumption was between 4 and 16 h. As expected, xylose and arabinose were not consumed during the fermentation and the acetic acid concentration was very low, reaching the value of 0.88 g/L. The results showed that the raw hydrolysate of starch from avocado seeds provided sufficient sugars to be fermented to ethanol without requiring additional supply nutrients and without producing inhibitory compounds such as furans.

### Ethanol fermentation by Saccharomyces cerevisiae at pilot scale

**Fig. 3:**
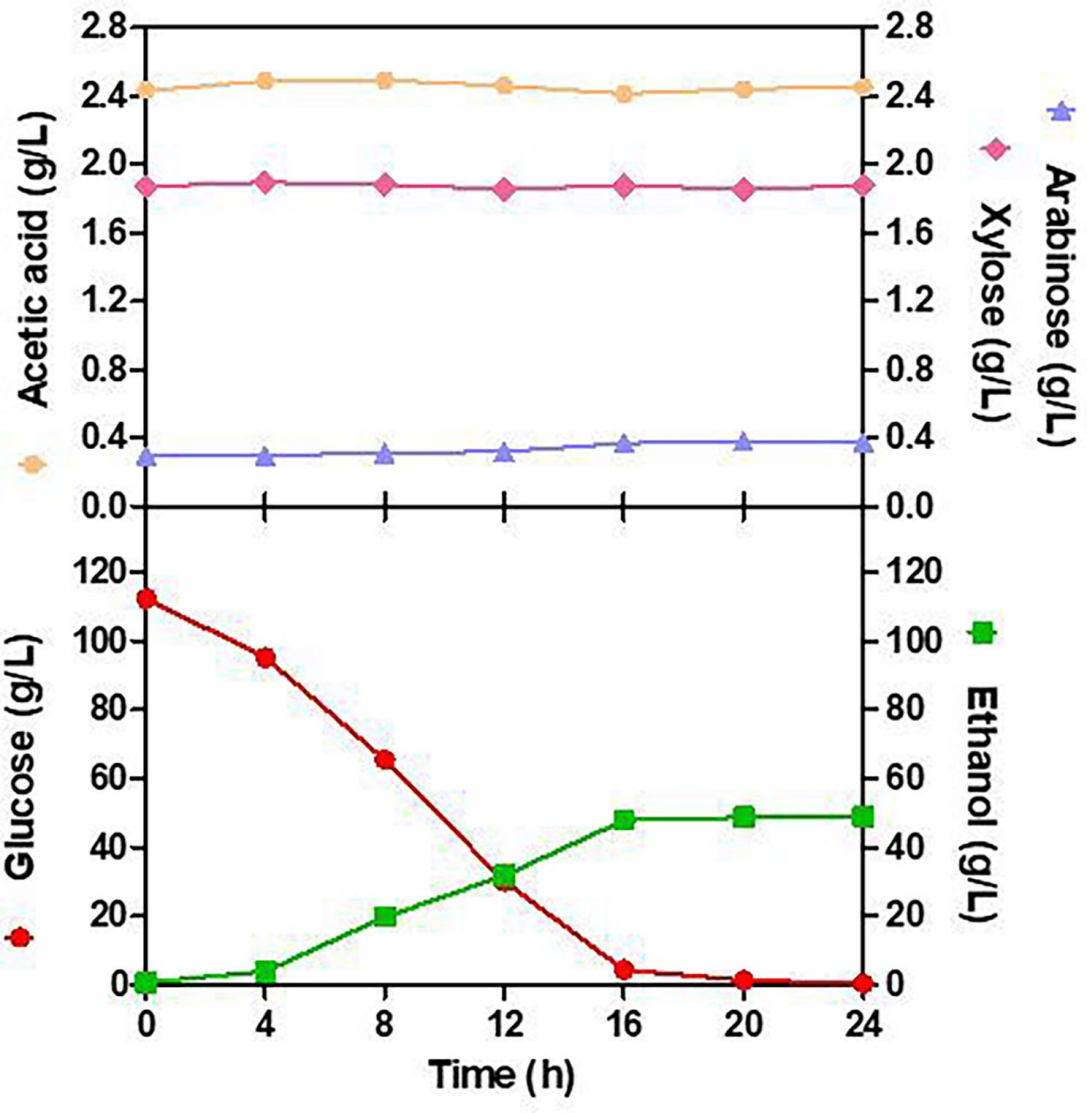
Fermentation kinetics of glucose obtained from the dilute acid pretreatment by *Saccharomyces cerevisiae* at pilot scale

**Fig. 4:**
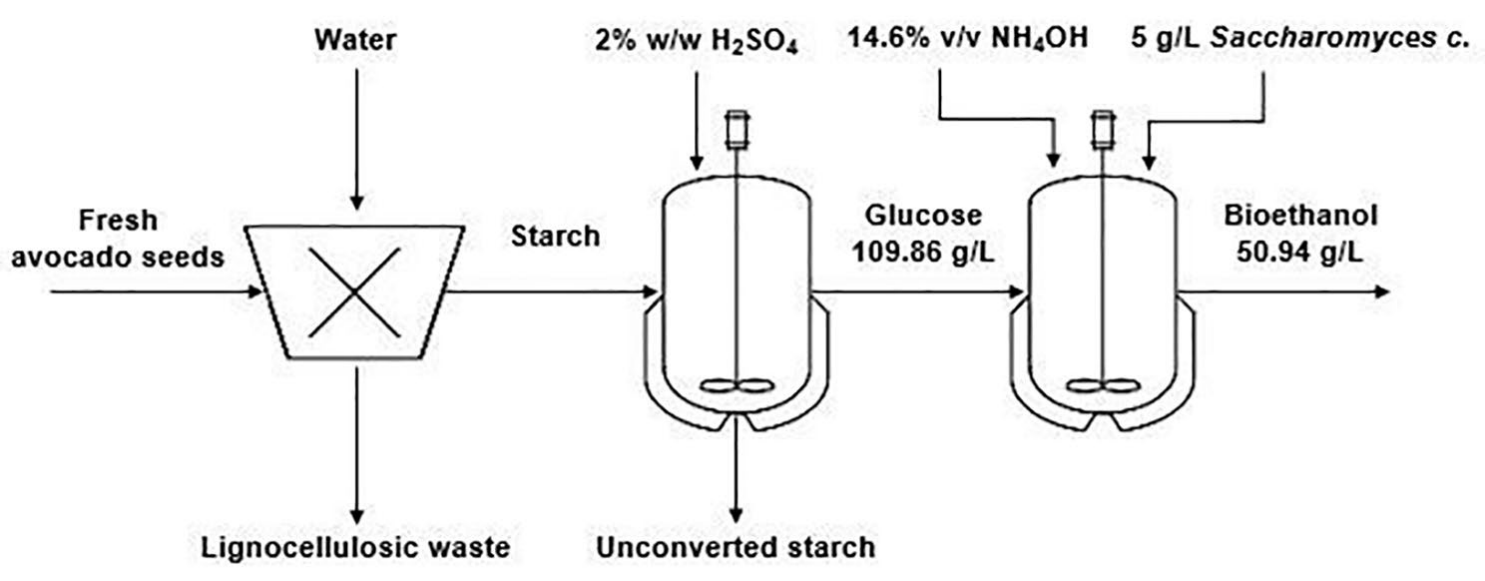
Flow diagram of the bioethanol production process under the proposed operating conditions (pilot scale). The proportion of ASs to water in the extraction starch was set at 1:3 (w/v) with a yield of 29.79%; acid hydrolysis efficiency was 86.38% at 87 °C and 12 h; the kinetic parameters of glucose fermentation to ethanol were: *p*_max_ = 50.94 g/L (6.46% v/v), *Y*_*p/s*_*=* 0.45 g_Ethanol/_g_Glucose_, *r*_*p*_*=* 2.11 g/L/h, and Ef = 88.74% at 30 °C and 24 h

The main goal of this scaling-up bioprocess is to check if the fermentation yield is maintained with the possibility, in the future, to be semi-industrializing bioethanol production using avocado wastes-derived fermentable sugars, the same parameters have been tested during ethanol fermentations carried out on a pilot scale (40 L). As shown in Fig. 3, the kinetics of yeast growth during fermentation in a pilot plant is very similar to occurred in laboratory-scale fermentations. The ethanol production and reduced sugar consumption for the two scales can be observed in Figs. 2 and 3. Small differences observed between fermentations were carried out in flasks and pilot plant, which may be due to differences in the agitation power and geometry of each system [72–74]. In fermentations carried out in the pilot plant, glucose was almost completely consumed at around 16 h and maximum ethanol concentration was also achieved at the same time. In the pilot case, ethanol fermentation finished around 24 h. As expected, the process was faster, since cell concentrations were a little bit higher as previously observed [74]. The values of *p*_max,_*Y*_*p/s*_, *r*_*p*_, and efficiency of the 40-L scale were at 50.94 g/L (6.46% v/v), 0.45g_Ethanol/_g_Glucose_ 2.11 g/L/h and 88.74%, respectively. Because of using raw starch, major by-products, i.e., acetic acid in the two scales were very low, in ranges of 0.88–2.45 g/L, and lactic acid was not produced, which is less than those values in the industries. The exponential phase for sugar consumption was also between 4 and 16 h. As expected, xylose and arabinose were also not consumed during the fermentation. As in laboratory fermentation assays, the results in the pilot-scale batch fermentation also showed that the raw hydrolysate of starch from avocado seeds provided sufficient sugars to be fermented to ethanol without requiring additional supply nutrients and without producing inhibitory compounds such as furfural and 5-hydroxymethyl furfural, after hydrolysis of starch from avocado seeds. As result, these hydrolysates can be further used for large-scale hydrolysis using other microorganisms for the production of commercial bioproducts (Fig. 4).

## Conclusion

A sequential hydrolysis and fermentation process of two scales for the ethanol production process from the starch of avocado seeds was conducted in this study (Fig. 4). Laboratory assays and pilot-plant studies demonstrated that ethanol fermentation from the starch hydrolysate with an initial concentration of glucose 112.44 ± 1.14 g/L was successful. The results of the present study also demonstrated that starch hydrolysate rich in glucose units can be fermented to ethanol without requiring an additional supply of nutrients into pretreated slurries. The significance of this work is that it includes the use of cheap chemicals for starch extraction and hydrolysis methods and the use of a natural *Saccharomyces cerevisiae* strain, which in turn makes the bioprocess of production of ethanol cost-effective. Furthermore, the laboratory process was technically quite easily feasible to scale up to a pilot scale with promising results. From the results, it could be concluded that the starch of avocado seeds is an attractive raw material for the production of bioethanol and can also be used for large-scale hydrolysis using other microorganisms for the production of other commercial bioproducts.

## Data Availability

The datasets used and/or analyzed during the current study are available from the corresponding authors on reasonable request.
